# The Potential of Topoisomerase Inhibitor-Based Antibody–Drug Conjugates

**DOI:** 10.3390/pharmaceutics14081707

**Published:** 2022-08-16

**Authors:** Seungmin Han, Kwang Suk Lim, Brody J. Blackburn, Jina Yun, Charles W. Putnam, David A. Bull, Young-Wook Won

**Affiliations:** 1 Division of Cardiothoracic Surgery, Department of Surgery, University of Arizona College of Medicine—Tucson, Tucson, AZ 85724, USA; 2 Department of Biotechnology and Bioengineering, College of Art, Culture and Engineering, Kangwon National University, Chuncheon 24341, Korea; 3 Department of Smart Health Science and Technology, College of Art, Culture and Engineering, Kangwon National University, Chuncheon 24341, Korea; 4 Department of Medical Pharmacology, University of Arizona College of Medicine—Tucson, Tucson, AZ 85724, USA; 5 Division of Hematology-Oncology, Department of Medicine, Soonchunhyang University Bucheon Hospital, Bucheon 14584, Korea

**Keywords:** topoisomerase inhibitor, topoisomerase 1, antibody–drug conjugates, targeted drug delivery, anti-cancer drug

## Abstract

DNA topoisomerases are essential enzymes that stabilize DNA supercoiling and resolve entanglements. Topoisomerase inhibitors have been widely used as anti-cancer drugs for the past 20 years. Due to their selectivity as topoisomerase I (TOP1) inhibitors that trap TOP1 cleavage complexes, camptothecin and its derivatives are promising anti-cancer drugs. To increase accumulation of TOP1 inhibitors in cancer cells through the targeting of tumors, TOP1 inhibitor antibody–drug conjugates (TOP1-ADC) have been developed and marketed. Some TOP1-ADCs have shown enhanced therapeutic efficacy compared to prototypical anti-cancer ADCs, such as T-DM1. Here, we review various types of camptothecin-based TOP1 inhibitors and recent developments in TOP1-ADCs. We then propose key points for the design and construction of TOP1-ADCs. Finally, we discuss promising combinatorial strategies, including newly developed approaches to maximizing the therapeutic potential of TOP1-ADCs.

## 1. Introduction

The DNA topoisomerase enzymes are pivotal for cell function and are found ubiquitously in all domains of life [1,2,3,4]. The various topoisomerase enzymes have roles in a wide range of functions related to the maintenance of DNA topology during DNA replication and transcription [1,2,3]. Topoisomerases support the fidelity of DNA replication and transcription by preventing or correcting topological problems, such as torsion, that may arise in double-helical DNA during its biochemical manipulation in the process of DNA replication or transcription [5,6,7].

Topoisomerase cleavage complexes (TOPcc), in which topoisomerases are bound to DNA breaks, are integral to topoisomerase-mediated changes in DNA topology but, importantly, also pose potential threats to genomic integrity [2,3]. For example, trapping of a TOPcc in advance of the replication machinery or during chromosome segregation, where interwoven DNA helices are unlinked by topoisomerases, can have adverse effects on genomic stability and, hence, cell viability [8]. Topoisomerase inhibitors act by stabilizing DNA–topoisomerase complexes, leading to double-strand breaks [2,3,4]. Among the six human topoisomerases (TOP1, TOP1MT, TOP2a, TOP2b, TOP3a, and TOP3b), TOP1 is essential to genomic stability due to its removal of both positive and negative DNA supercoils that might otherwise lead to DNA breaks [3,4].

Topoisomerase 1 inhibitors have been investigated clinically and several have been granted FDA approval [9,10]. Obstacles, such as chemical instability and short plasma half-lives, hinder their efficacy and contribute to side effects [2,4,11]. Consequently, tumor-targeted drug delivery strategies, such as antibody–drug conjugates (ADCs), are in clinical development to overcome these limitations [4,12]. Here, we review the importance of TOP1 inhibitor-based ADCs (TOP1-ADCs) in cancer therapy, the current state of knowledge regarding the design of TOP1-ADCs, and TOP1-ADCs under development. We then discuss perspectives on combinatorial strategies with other agents to maximize the potential benefit of TOP1-ADCs in cancer treatment.

## 2. TOP1 Inhibitors in Cancer Therapy

**Figure 1:**
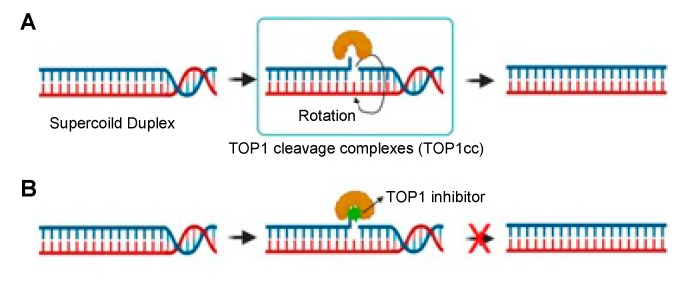
TOP1 and TOP1 inhibitor mechanism. (**A**) TOP1 forms 3′-phosphotyrosine bonds, allowing the cut DNA strand to rotate about the intact one, thus relaxing DNA supercoiling. (**B**) A TOP1 inhibitor binds at the TOP1 enzyme–DNA interface to prevent DNA re-ligation and to lock the enzyme into TOP1cc. The TOP1 inhibitor thus generates DNA damage and, ultimately, cell death.

TOP1 is a conserved enzyme essential for relaxing supercoiled DNA to alleviate helical constraints (Figure 1A). DNA supercoiling is important to pack DNA within cells; it is generated by the unwinding of double-stranded DNA (dsDNA) by DNA or RNA polymerases during DNA replication or at sites of transcription [2]. TOP1 binds to supercoiled dsDNA and cleaves one DNA strand, allowing relaxation of the supercoil by rotation; TOP1 also aids in the reannealing process. When TOP1 is inhibited, supercoils halt replication forks or transcription complexes, blocking both processes and potentially causing dsDNA breaks [2,4,13].

**Figure 2:**
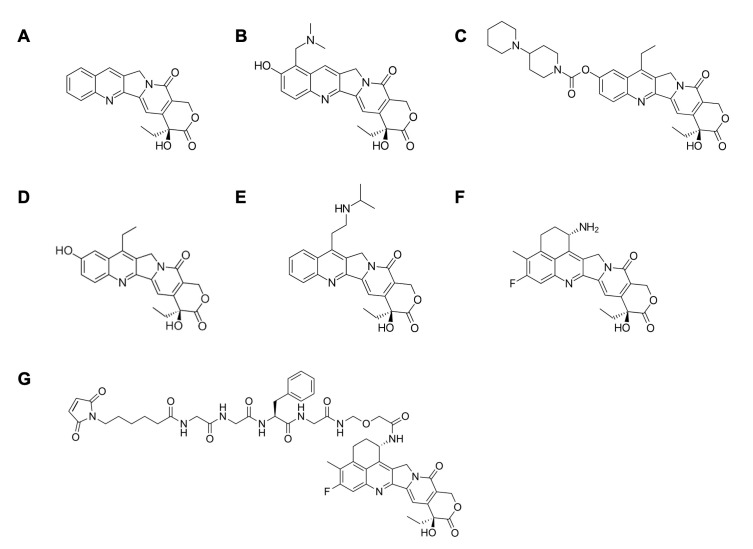
Chemical structure of (**A**) camptothecin, and its derivatives: (**B**) topotecan, (**C**) irinotecan, (**D**) SN-38, (**E**) belotecan, (**F**) exatecan, and (**G**) deruxtecan.

Camptothecin is a TOP1 inhibitor produced by nature [4]. TOP1 inhibitors work by binding to TOP1cc (see Figure 1B). The selective binding of camptothecin at the TOP1–DNA interface leads to its classification as an “interfacial inhibitor”. This class of inhibitors binds to the interface of macromolecules; thus, the TOP1cc provides a specific binding site for camptothecin at the junction of TOP1 and DNA [14]. It has been shown that camptothecin and its derivatives are highly selective for such macromolecular complexes; TOP1cc is the sole target of these agents. Therefore, it has been argued that camptothecin is an ideal pharmacological agent as its selectivity, rather than potency, dictates its therapeutic effect [8]. Recently, camptothecin-derived TOP1 inhibitors, such as topotecan, irinotecan, belotecan, and deruxtecan, have been approved for the treatment of NSCLC, ovarian cancer, and colorectal cancer (Figure 2) [15,16,17].

Of the several camptothecin derivatives poised to become pharmacological agents in various cancer treatment protocols, exatecan shows great promise; it is 6 and 28 times more active than SN-38 and topotecan, respectively [18]. In a study published in *Cancer Chemotherapy and Pharmacology*, the therapeutic effects of exatecan mesylate, in its salt form (DX-8951f), were compared to those of other FDA-approved camptothecin derivatives, such as irinotecan (CPT-11) and topotecan (SK&F104864), using human tumor xenografts in nude mice [19]. A total of 16 human cancer lines were examined: six colon cancer, five lung cancer, two breast cancer, one renal cancer, and two gastric cancer cell lines. The two gastric cancer cell lines included a CPT-11-sensitive tumor, gastric adenocarcinoma SC-6, and its CPT-11-resistant variant, SC-6/CPT-11. After a total of four injections four days apart, the tumor growth inhibition rate (IR) of exatecan was ≥58% in 15 of the 16 cancer cell lines and ≥80% in 14 of them. By contrast, CPT-11 displayed an IR ≥58% in only 11 of the cell lines and an IR ≥80% with just 8 of the 16 cell lines. In addition, exatecan was judged effective against the gastric line SC-6/CPT-11, whereas CPT-11 was not. In summary, the study indicated that exatecan has superior antitumor activity against a panel of cancer cell lines and over a broader range of doses than other camptothecin derivatives, such as irinotecan (CPT-11) and topotecan (SK&F104864) [19]. Importantly, many chemotherapeutics are rendered ineffective by multidrug resistance executed by P-glycoprotein (P-gp)-mediated cellular efflux. Exatecan, however, may overcome P-gp-mediated drug resistance because it appears to be an incompatible substrate for this membrane drug transporter [20]. Despite these favorable characteristics, exatecan remains in phase III clinical trials and has not yet been clinically approved because of its significant myelotoxicity [21]. Gastrointestinal toxicity and bone marrow toxicity are the typical limiting factors in the clinical use of TOP1 inhibitors [4]. However, deruxtecan (Dxd), an exatecan derivative, has similar TOP1 inhibitory effects yet lower myelotoxicity than exatecan [22]. Thus, the former has been utilized in the development of TOP1-ADCs with greater safety, such as DS-8201a (trastuzumab–Dxd), which has been approved by the FDA for the treatment of breast cancer [23]. In addition, DS-8201a inhibited the growth of the T-DM1 resistant cell line N87-TDMR. As cancer patients can acquire DM1 resistance due to DM1′s compatibility with the membrane drug transporter and the consequent increased efflux of DM1 from cells, TOP1 inhibitors are arising as potent drugs for cancer patients who have developed resistance to drugs that are substrates for the membrane drug transporter [24].

## 3. Importance of Targeted Delivery of TOP1 Inhibitors: TOP1-ADC

Surgery and/or radiotherapy are primary therapies for localized tumors, while metastases are usually treated with chemotherapeutic regimens. Traditional chemotherapeutics are nonspecific in terms of targeting tumor cells versus normal cells; they exhibit some selectivity for rapidly dividing malignant cells because chemotherapeutic agents most often target the cell cycle. When chemotherapeutic drugs are delivered by nonspecific targeting, higher doses are required to eradicate tumors, which in turn may lead to dose-limiting side effects [25].

**Figure 3:**
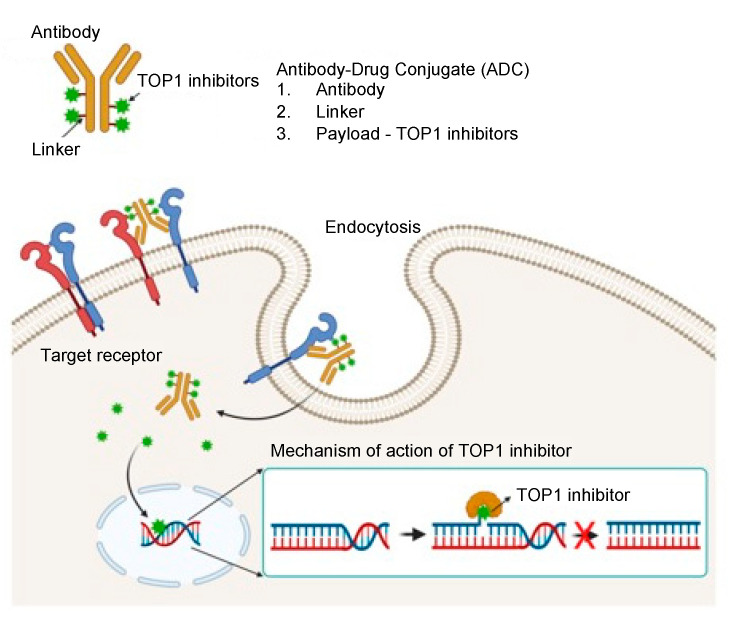
Schematic illustration of the therapeutic mechanism of TOP1-ADC.

Although TOP1 inhibitors effectively induce cancer cell death, their potential applicability is limited by: (i) their rapid elimination before achieving therapeutic concentrations in the tumor; and (ii) dose limitations dictated by the inhibition of TOP1 in normal cells, which need topoisomerase activity for cell survival. To increase the tumor-specific delivery of TOP1 inhibitors, and hence their accumulation in cancers, TOP1-ADCs and nanoparticulate formulations have been developed; the latter include liposomes, polymeric nanoparticles, and functionalized carbon nanotubes [26,27,28,29,30]. Nanoparticulate formulations increase drug solubility and extend in vivo half-life; however, because of their passive targeting effect—namely, enhanced permeability and retention (EPR)—their tumor-targeting efficiency remains problematic. Although the EPR effect improves tumoral delivery of nanoparticles, their efficiency of delivery to the tumor remains less than one percent of the injected dose of nanoparticles [31]. With TOP1-ADCs, however, monoclonal antibody-mediated active targeting offers both selectivity and extremely high affinity because of specific antibody–antigen binding, which can distinguish tumor versus healthy cells based on antigen expression levels [32]. Consequently, TOP1-ADC is an effective approach to enhance the anti-tumor activity of both the monoclonal antibody and the TOP1 inhibitor (Figure 3). ADCs are designed to deliver TOP1 inhibitor specifically to tumor cells without an off-target effect. Moreover, conjugation of the cytotoxic agent to the large, hydrophilic antibody protein is predicted to restrict the penetration of the cytotoxic compound across the cellular membranes of antigen-negative normal cells, further lessening off-target side effects [33].

## 4. Optimization Strategies for TOP1-ADC

To enhance the efficacy of TOP1-ADC for cancer therapy, all three components of an ADC must be optimized: the targeting antibody, the linker connecting drug to antibody, and the TOP1 inhibitor. Additionally, antigen selection and the drug-to-antibody ratio (DAR) are important considerations in designing an effective ADC [33,34].

### 4.1. Selection of Antibody Type and Target Antigen

Monoclonal antibodies (mAbs) offer extremely high affinity and selectivity for cell surface antigens, which can differentiate tumor cells versus healthy cells based on the relative expression levels of the antigen. Immunoglobulin G (IgG) is the dominant antibody backbone for ADCs. Among the human IgG subclasses (IgG1, IgG2, IgG3, and IgG4), IgG1 antibodies have greater stability—and hence a longer serum half-life—and higher Fcγ receptor-binding efficiency, increasing their innate immune activity (Table 1) [35]. Consequently, IgG1 is most often selected as the ADC backbone. 

Structurally, the antibody has two regions, Fc and Fab: the Fc fragment binds to the Fcγ receptor, and the Fab fragment binds to the target antigen. Fab is therefore designated as the variable region, in which the *variable heavy* and *variable light* domains (V_H_ and V_L_, respectively) pair to form the antigen-binding site of the antibody [36]. The Fab portion of the antibody delivers the ADC to the cancer cell, rendering the ADC a targeted therapy. However, for high therapeutic efficacy yet low off-tumor, on-target effects, the target antigen and, therefore, the relevant Fab of the antibody must be strategically selected. HER2, TROP2, CEACAM5, B7-H3, and nectin4 are examples of target antigens for ADCs that have been approved for the treatment of solid tumors [37]. Additionally, the HER3 antigen is currently under investigation as a tumor-specific antigen for ADC designed to treat metastasized brain cancer or HER2-resistant metastases [38,39,40,41]. Thus, the repertoire of TOP1-ADCs, both in clinical trials and already FDA-approved, target HER2, HER3, TROP2, CEACAM5, and B7-H3.

In addition to tumor-specific antigenicity, homogeneity of antigen expression is a second consideration. For example, breast cancers with a high level of heterogeneity in HER2 expression respond poorly to T-DM1 when compared to those with homogenous HER2 expression [42]. Selective targeting of functionally oncogenic antigens has the additional benefit of antibody-mediated suppression of downstream oncogenic signaling pathways [43,44]. The search continues for new targets of novel ADCs that could provide better treatment approaches [45].

### 4.2. Selection of Linkers 

**Figure 4:**
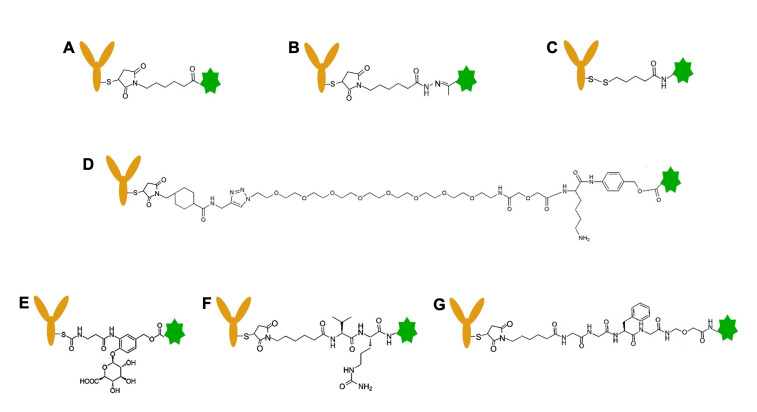
Chemical structures of linkers for antibody–drug conjugation. (**A**) SMCC linker, (**B**) hydrazone linker, (**C**) disulfide linker, (**D**) CL2A linker, (**E**) β-glucuronide linker, (**F**) Val-Cit (VC) linker, (**G**) Gly-Gly-Phe-Gly (GGFG) linker. (**A**) is a non-cleavable linker, and (**B**–**G**) are cleavable linkers. Green- and brown-colored icons represent TOP1 inhibitor and antibody, respectively.

A vital component of an ADC is the linker that constitutes the connection between payload and antibody. Choosing the preferred crosslinker is crucial not only to provide stability for the ADC but also to assure optimal release of the payload at the desired location [33]. Both the type of crosslinker and the location of conjugation can affect the pharmacokinetics, efficacy, and clinical tolerance of the ADC. The mechanism for drug release is also dependent on the type of linker, be it cleavable or non-cleavable (Figure 4). Examples of cleavable crosslinkers include: acid-sensitive linkers, glutathione-sensitive linkers, lysosomal protease-sensitive linkers, and β-glucuronide linkers [46].

A non-cleavable crosslinker is said to offer greater plasma stability because of its non-reducible amino acid linker and thioether bond, which reduces non-specific drug release compared to ADCs with cleavable linkers [47]. With a non-cleavable ADC, the drug is dissociated from the antibody after internalization and lysosomal proteolytic degradation of the antibody [33,48]. The first generation of FDA-approved ADCs employed an acid-cleavable hydrazone linker, which makes use of the lower pH of endosomal and lysosomal compartments. The linker is stable in blood at neutral pH; once the ADC is internalized into acidic endosomes and lysosomes (pH = 4–6), the linker is cleaved. The release of the drug after 24 h at 37 °C is 97% at pH = 4.5, whereas only 6% was freed at pH = 7.4 [49]. The pH-sensitive linkers include hydrazone linkers, disulfide linkers, and the CL2A linker. Disulfide linkers are cleavable by both pH and glutathione [50]. The enzymatically cleavable linkers, such as the β-glucuronide linker, valine-citrulline (VC)-based linker, or glycyl-glycyl-phenylalanyl-glycyl (GGFG) linker, are cleaved by lysosomal proteases. TOP1-ADCs have utilized both the CL2A and the GGFG linkers. Chemical, near-infrared light, and tumor microenvironment-triggered payload-release strategies have also shown promising results experimentally [51,52,53,54].

Every linker has advantages and disadvantages; there is no single, optimal linker for all ADCs. Each pair of antibodies and drugs must also be optimized to obtain the best therapeutic outcome. New linkers can be designed to improve water solubility (e.g., PEGylation) and tumor selectivity (e.g., enzymatic-cleavable, photocleavable, proton cleavable) [55,56,57,58]. 

### 4.3. Optimization of Drug-to-Antibody Ratio (DAR)

The DAR is the average number of payloads attached to each antibody, and it affects both the stability and drug activity of the ADC [33,37,38]. If the mAb is heavily conjugated—that is, it has a high DAR—the hydrophilicity, biodistribution, and pharmacokinetics of the ADC are substantially affected [59,60]. It has been shown that the in vivo cytotoxicity of an ADC with heavily loaded antibodies (high DAR) is decreased because of enhanced plasma clearance, primarily by cells in the liver, such as hepatic sinusoidal endothelial and Kupffer cells [59]. Therefore, most current ADCs have average DARs of two to four in order to retain hydrophilicity and avoid aggregation [33,61,62]. However, as hydrophilicity-enhancing linker technologies are developed, ADCs with higher DAR may be practicable.

To overcome the DAR 4 barrier, Daiichi Sankyo has successfully developed the deruxtecan ADC franchise (DS-8201a, U3-1402) using a newly-developed hydrophilic linker (GGFG) designed to reduce the hydrophobicity and aggregation of the ADCs [33,63,64,65,66]. TOP1-ADCs, such as DS-8201a and U3-1402, have DAR 8 using the GGFG linker. It is known that high DAR typically induces ADC instability and quick clearance, but the GGFG linker lends hydrophilicity to the ADC, resulting in higher stability and slower clearance of the ADC [67,68]. Consequently, they have achieved DAR 8, with greater stability in plasma, slower in vivo drug clearance, and reduced toxicity due to off-target cytotoxicity from liberated chemotherapeutic agents in the blood [63]. High DAR directly impacts the anti-tumor activity of TOP1-ADC. Consequently, DS-8201a should have stronger therapeutic efficacy in HER2-positive cancer than Kadcyla (T-DM1), which is a commonly used ADC for HER2-positive cancer [69,70]. Although there are likely other properties contributing to the stronger therapeutic efficacy of DS-8201a over that of T-DM1, high DAR is a straightforward concept by which to increase therapeutic efficacy [63]. Various efforts in drug-linker design are currently ongoing to generate ADCs with high DAR values without negatively impacting their pharmacological properties [71,72,73]. 

### 4.4. Linkage Strategy to Generate TOP1-ADC

The amine group (-NH_2_) of lysine and sulfhydryl group (-SH) of cysteine in an antibody are generally available for attachment of the linker. A typical IgG1 antibody has roughly 80 lysine residues, but fewer than ten are chemically available for modification. Although random conjugation to lysine residues may generate higher DAR, targeting lysine generates a heterogeneous ADC with a spectrum of DARs and differing conjugation sites. A heterogeneous ADC population with random conjugation sites may confer suboptimal potency and altered solubility, stability, and/or pharmacokinetics, all affecting their antigen-binding properties. Conversely, the advantage of cysteine-mediated conjugation is that the limited number and locations of cysteine-conjugation sites generate lower heterogeneity than lysine-based conjugation. Cysteines have a role in the intrachain and interchain disulfide bridges of antibodies. IgG1 antibody has four interchain disulfide bridges, and the bridges can be reduced to eight sulfhydryl groups for conjugation. TOP1-ADCs in a clinical trial for FDA approval have utilized the sulfhydryl group of cysteine to generate ADC. Maleimide chemistry is a common linker strategy for sulfhydryl conjugation. One limitation of maleimide chemistry is that its instability in plasma allows separation of drug-linkers from the ADC. Therefore, work is underway to improve the stability of sulfhydryl-based conjugation; for example, by hydrolysis of succinimide [74,75].

**Figure 5:**
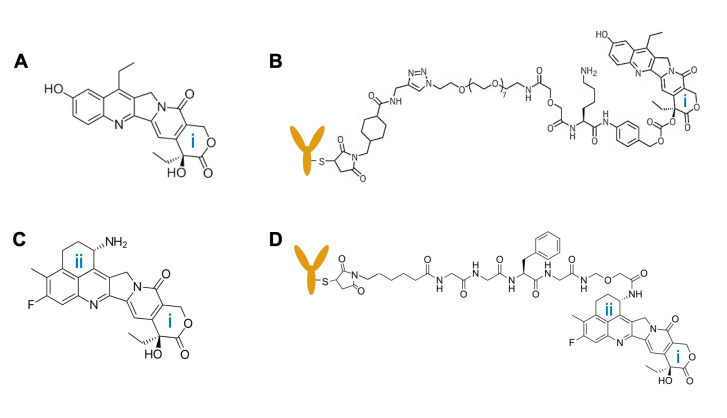
Conjugation sites of TOP1 inhibitors for linker attachment and the chemical structure of the cognate TOP1-ADC. (**A**) hydroxyl group in (i)-ring of SN-38 structure, (**B**) SN-38-based TOP1-ADC, (**C**) hydroxyl group in (i)-ring and amine group in (ii)-ring of exatecan, and (**D**) exatecan derivate (Dxd)-based TOP1-ADC. The antibody is depicted as a brown-colored icon.

A major concern in joining the linker to the camptothecin-derived drug is to avoid conjugation sites that diminish drug activity. Since TOP1-ADCs in current clinical trials are formulated with SN-38 or Dxd (exatecan derivative), we focus upon these two drugs in our discussion of linker conjugation. Camptothecin derivatives typically have at least one functional group available for conjugation: the hydroxyl group of the (i)-ring and/or the amine group of the (ii)-ring (see Figure 5). It is known that the open form of the (i)-ring renders the drug less active. Therefore, the (i)-ring of SN-38 was stabilized in order to keep the closed form of the (i)-ring intact after linker–hydroxyl group conjugation for the generation of SN-38-mediated TOP1-ADC [21]. After exatecan was developed, it was observed that its (ii)-ring stabilizes the structure of the (i)-ring, leading to greater stability and increased cytotoxicity in the drug [21]. Thereby, the exatecan derivative (Dxd) could be conjugated using the amine group of the (ii)-ring without the necessity of further stabilizing the (i)-ring, a strategy applied to TOP1-ADC. Thus, choosing the appropriate linker and binding sites for both the antibody and the drug are important strategic decisions when designing and generating stable and effective TOP1-ADCs.

## 5. TOP1-ADC in Clinical Trial/FDA Approval

Rapid developments in conjugating TOP1 inhibitors to monoclonal antibodies mirror the direction of modern target-based therapies (Table 2). SN-38 has been utilized to generate IMMU-132 and IMMU-130 ADCs using the CL2A linker. The cytotoxic payload includes the active metabolite of irinotecan, SN-38. Irinotecan (CPT-11) must be hydrolyzed by carboxylesterase to produce the active metabolite SN-38, which confers cytotoxic activity [4,65]. IMMU-132 (sacituzumab govitecan) is an ADC in which a humanized anti-TROP2 monoclonal antibody is conjugated to SN-38 via a cleavable crosslinker. The linker promotes the dissociation of the payload from the antibody in lower pH environments, such as that of lysosomes [65]. The transmembrane glycoprotein TROP2 is commonly overexpressed in many epithelial cancers [4,65]. IMMU-132 was recently approved by the FDA for metastatic triple-negative breast cancer (TNBC) [76]. IMMU-130, labetuzumab govitecan, is a CEACAM5-targeting ADC utilizing CL2A-SN-38 [66,77]. The conjugate is currently in a phase I/II clinical trial for the treatment of colorectal cancer. IMMU-140 is a novel SN-38 ADC containing a humanized anti-HLA-DR IgG4 antibody (IMMU-114) that is devoid of immune function [64]. HLA-DR is a MHC class II antigen and is expressed by hematological and solid tumors [64]. 

The exatecan derivative deruxtecan (Dxd) is more potent than irinotecan in several in vivo tumor xenograft models, including irinotecan-resistant tumors [68]. Dxd-mediated ADCs in clinical trials include DS-8201a, U3-1402, DS-1062, and DS-7300a [3,4]. All four ADCs use a peptide linker that is susceptible to cleavage by lysosomal proteases [63]. The antibodies conjugated with Dxd in the DS-8201a, U3-1402, DS-1062, and DS-7300a ADCs are, respectively, anti-HER2, anti-HER3, anti-TROP2, and anti-B7-H3 [39,63,78]. They are in clinical trials for breast cancer and lung cancer [39]. In a breast cancer patient-derived xenograft (PDX) model, DS-8201a (DAR 8) at a dose of 10 mg/kg exhibited impressive antitumor activity against a low HER2-expressing tumor, while T-DM1 (DAR 3.5) displayed virtually no cytotoxicity against the same tumor at similar doses [63]. Similar observations were made when using a pancreatic ductal adenocarcinoma mice xenograft model, Capan-1, which is likewise a weakly HER2-positive carcinoma; DS-8201a displayed exceptional tumor cytotoxicity, while T-DM1 was largely ineffective [63]. Most importantly, DS-8201a was shown to have impressive therapeutic efficacy in cancer patients with HER2-low and HER2 heterogeneous breast cancer [79]. The superior performance of DS-8201a compared to T-DM1 may be attributable, at least in part, to its considerably higher DAR of 8, which arguably enhances its cytotoxic effects in tumor cells that express HER2 only at low levels [80]. The cytotoxic profile of DS-8201a and other topoisomerase inhibitor-based ADCs appear potentially superior to ADCs containing microtubule-targeting payloads and should therefore be considered in cases where microtubule-inhibiting conjugates are no longer effective.

## 6. Suggestions for Improving Therapy with TOP1-ADC

Although the efficacy of cancer therapy with TOP1 inhibitors has been greatly enhanced with the TOP1-ADCs, there remains ample room for improvement in the therapeutic efficacy of the TOP1-ADC conjugates. In this section, we review the combinatorial candidates with which to enhance the therapeutic efficacy of TOP1-ADCs (Table 3).

### 6.1. DNA Damage Response Modulators

Poly(ADP-ribose)polymerase (PARP) inhibitors are FDA-approved drugs for various cancers. PARP promotes DNA stabilization and repair; consequently, PARP inhibitors halt DNA stabilization and, therefore, are predicted to sensitize cancer cells to TOP1 inhibitors [81]. There is clinical research underway to investigate the possible synergistic effects of a PARP inhibitor and a TOP1-ADC (DS-8201a: NCT04585958 and IMMU-132: NCT04039230).

The inhibition of cell cycle checkpoint proteins, such as ataxia telangiectasia mutated and Rad3-related kinase (ATR) and its major downstream effector checkpoint kinase 1 (CHK1), is another strategy to increase the anti-cancer efficacy of TOP1-ADC.

DNA damage activates ATR and CHK1 to protect cells by arresting cell cycle progression and providing time for repair and recovery [82,83]. Inhibition of ATR or CHK1 increases the sensitivity of cancer cells to TOP1 inhibitors [3]. Recent studies have examined ATR inhibitors (M6620 and AZD6738) in combination with TOP1 inhibitors (topotecan/irinotecan). A phase I trial of M6620/irinotecan combination (clinicaltrials.gov identifier: NCT02595931) and a phase II trial of M6620/topotecan (NCT 02487095) in lung cancer are underway. The combination of DS-8201a and AZD6238 is in a phase I trial to test possible therapeutic efficacy against 75 types of advanced solid tumors (NCT04704661). Additionally, the combination of IMMU-132 and M6620 is under evaluation in an ongoing phase I/II trial (NCT04826341). However, a clinical trial of the combination of CHK1 and TOP1 inhibitors was terminated because of off-target cardiac toxicity of the CHK1 inhibitor [84,85]. Based on the latter finding, we hypothesize that inhibiting DNA damage response molecules might act synergistically with a TOP1-ADC. A multi-drug ADC might be a solution to simultaneously targeting tumors with DNA damage response and TOP1 inhibitors. Creating multi-drug ADC by using unmasking of cysteine residues combined with orthogonal protection enables site-specific conjugation of each drug, as reported by Levengoo M. et al. [86]. This approach would strengthen tumor-specific therapeutic effects while protecting patients from off-target side effects.

### 6.2. Immunotherapy

Immunotherapy has attracted much attention as a candidate for combinatorial therapy with TOP1-ADC. TOP1 inhibitor-mediated DNA damage can stimulate both innate and adaptive immune responses through multiple mechanisms: (1) release of DNA fragments after DNA damage, which activates the stimulator of interferon genes (STING) pathway [87,88,89]; (2) DNA damage-induced release of tumor microvesicles, which increase immune activation [90,91]; and (3) DNA damage-induced antigen presentation via tumor cell MHC class I molecules, which enhance recognition of tumor cells by immune cells [87,88,89,90,91,92]. Indeed, a synergistic anti-cancer effect against HER2-expressing cancer cells was observed when irinotecan or DS-8201a was combined with an anti-PD-1 antibody [67,93]. The authors speculate that upregulated expression of MHC class I and PD-L1 induced by DS-8201a was the basis for the synergistic effect of the combination [67]. This combination is in phase I/II trials for patients with breast cancer or NSCLC (NCT03523572 and NCT03334617).

Another approach to combining TOP1-ADC with immunotherapy is to use TOP1-ADC to arm immune cells. There are efforts underway to develop engineered immune cells that combine both immunotherapy and chemotherapy with a tumor targeting capability [94,95]. Lee et al. have developed ADC-mediated surface-engineered natural killer (SENK) cells [94]. ADC is conjugated with a polyethylene-glycol-grafted lipid. Then, the ADC lipid is embedded in the NK cellular membrane to arm NK cells. Once SENK cells are generated, the antibody of the ADC directs the NK cells to cancer cells. Then, both the ADC and the NK cells cooperate in tumor cell elimination through the combination of ADC-mediated chemotherapy and NK cell-mediated immunotherapy. The group generated ADC-SENK with T-DM1 and demonstrated that T-DM1-SENK had improved anti-cancer activity, compared to NK cells alone, against HER2-positive tumors. In addition, Li et al. generated ADC-armed NK cells using a single-strand DNA (ssDNA) linker [95]. The ADC and NK cells were each modified with ssDNAs that had complementary sequences. ADC-armed NK cells showed higher therapeutic efficacy compared to bare NK cells or ADC itself. Although both studies employed T-DM1 to generate engineered NK cells, this concept can be expanded to TOP1-ADC and other immune cells. We suggest that TOP1-ADC-engineered immune cells may ultimately result in improved tumor treatment and improved survival rates. 

### 6.3. Establish Assays for Monitoring Topoisomerase Activity

The cytotoxic effect of topoisomerase inhibitors is positively correlated with the expression level and activity of topoisomerase [2]. Therefore, when treating patients with low expression levels or diminished activity of topoisomerase 1, TOP1-ADC might not have sufficient therapeutic efficacy, even when the conjugate is successfully targeted to the cancer cell. However, it is not known whether the correlation between topoisomerase levels and drug response is a simple linear relationship [96,97]. This limitation can be best addressed by monitoring the expression level and activity of topoisomerase before treatment. That would require identifying a molecular biomarker of the tumor that reflects topoisomerase activity and the DNA damage response [98].

## 7. Conclusions

TOP1 inhibitors have shown considerable potential as therapeutic agents against cancers; however, their clinical application has been hindered by their adverse pharmacokinetic profiles and off-target toxicities. Both obstacles can be mitigated by targeting their delivery via tumor-specific antibodies, as in TOP1-ADC formulations. The strategically chosen antibody must specifically and selectively target an antigen expressed on tumor cells; by using different tumor-targeting antibodies, the spectrum of tumor types is broadened. The crosslinker design should not only confer stability to the conjugate, minimizing premature drug release, but also allow for efficient release in the cancer cell. Additionally, conjugation of the linker to the antibody must not compromise its specificity nor should linker–drug conjugation degrade the desirable chemical properties of the payload. In turn, the cytotoxic payload, a TOP1 inhibitor, should be formulated to optimize both cytotoxicity and conjugation site(s). A final consideration in TOP1-ADC formulation is that higher drug-to-antibody ratios (DARs) may further improve their therapeutic efficacy. Further research on various anticancer drugs and cancer cell targets is needed to identify additional candidates for combination with TOP1 inhibitors. The development of multi-drug ADCs consisting of TOP1 and a second anticancer agent have considerable potential for synergistic anticancer effects, especially in tumors that have developed resistance to primary therapies.

## Figures and Tables

**Table 1:** Stability in serum and Fcγ receptor binding of human IgG subclasses.

IgG Subclass	IgG1	IgG2	IgG3	IgG4
**Serum half-life**	21 days	21 days	7–21 days	21 days
**Fc** **γ** **receptor binding**	High	Low	High	Moderate

**Table 2:** Camptothecin-derived TOP1-ADCs, either in clinical trials or FDA-approved, with their representative clinical trial identifiers. Abbreviations: TROP-2 (trophoblast cell surface antigen 2), CEACAM5 (carcinoembryonic antigen-related cell adhesion molecule 5), HLA-DR (human leukocyte antigen-DR), HER2 (human epidermal growth factor receptor 2), HER3 (human epidermal growth factor receptor 3), SN38 (7-ethyl-10-hydroxycamptothecin, irinotecan analog derived from camptothecin), Dxd (deruxtecan), NSCLC (non-small cell lung cancer), SCLC (small cell lung cancer).

ADC	Status	Antibody Target	Drug	Linkers	DAR	Target Cancer	Clinical Trial Identifier
Sacituzumab govitecan (IMMU-132)	FDA-approved	TROP2	SN38	CL2A (pH-sensitive cleavage)	7.6	Triple-negative breast cancer/urothelial cancer (phase II)	NCT04320693
Labetuzumab govitecan (IMMU-130)	Phase I	CEACAM5	SN38	CL2A (pH-sensitive cleavage)	7.6	Colorectal cancer	NCT01270698
Trastuzumab deruxtecan (DS-8201a)	FDA-approved	HER2	Dxd	GGFG (lysosomal enzyme cleavage)	8	Metastatic HER2-positive breast cancer	NCT03384940
Patritumab deruxtecan (U3-1402)	Phase I/II	HER3	Dxd	GGFG (lysosomal enzyme cleavage)	8	NSCLC (phase I) breast cancer (phase I/II)	NCT02980341, NCT03260491
DS-1062	Phase III	TROP2	Dxd	GGFG (lysosomal enzyme cleavage)	4	Breast cancer, NSCLC	NCT05104866, NCT05215340
DS-7300a	Phase I/II	B7-H3	Dxd	GGFG (lysosomal enzyme cleavage)	4	Solid cancer, SCLC	NCT05280470, NCT04145622

**Table 3:** Various strategies to enhance the therapeutic efficacy of TOP1-ADCs.

	Strategy	Effect
DNA damage response modulator	PARP inhibitor	Inhibit DNA stabilization and sensitize cancer cells to TOP1 inhibitor
	ATR/CHK1 inhibitor	Inhibit cellular recovery and increase the sensitivity of cancer cells to TOP1 inhibitor
Immunotherapy	Checkpoint inhibitor	Inhibit checkpoint molecule that is increased by topoisomerase inhibitor and enhance immunosurveillance
	ADC-mediated immune cell engineering	Dual effect: increase tumor targeting of immune cells and deliver a drug to cancer
Enzyme activity	Monitoring of topoisomerase activity	Increase drug response through selective treatment

## Data Availability

Not applicable.
